# A Protocol for FRET-Based Live-Cell Imaging in Microglia

**DOI:** 10.1016/j.xpro.2020.100147

**Published:** 2020-10-23

**Authors:** Renato Socodato, Pedro Melo, José P. Ferraz-Nogueira, Camila C. Portugal, João B. Relvas

**Affiliations:** 1 Instituto de Investigação e Inovação em Saúde and Instituto de Biologia Molecular e Celular, Universidade do Porto, 4200-135 Porto, Portugal; 2 Unit of Experimental Biology, Department of Biomedicine, Faculty of Medicine, University of Porto, 4200-319 Porto, Portugal

## Abstract

This protocol highlights the use of FRET-based biosensors to investigate signaling events during microglia activation in real time. Understanding microglia activation has gained momentum as it can help decipher signaling mechanisms underlying the neurodegenerative process occurring in neurological disorders. Unlike more traditional methods widely employed in the microglia field, FRET allows microglia signaling events to be studied in real time with exquisite subcellular resolution. However, FRET-based live-cell imaging requires application-specific biosensors and specialized imaging systems, limiting its use in *in vivo* studies.

For complete details on the use and execution of this protocol, please refer to Socodato et al. (2020), Portugal et al. (2017), and Socodato et al. (2018).

## Highlights

• A protocol for FRET-based live-cell imaging in microglia is described
• The FRET protocol can also be implemented for other CNS cell types
• FRET allows unprecedented real-time visualization of microglial signaling

## Graphical Abstract

**Figure:**
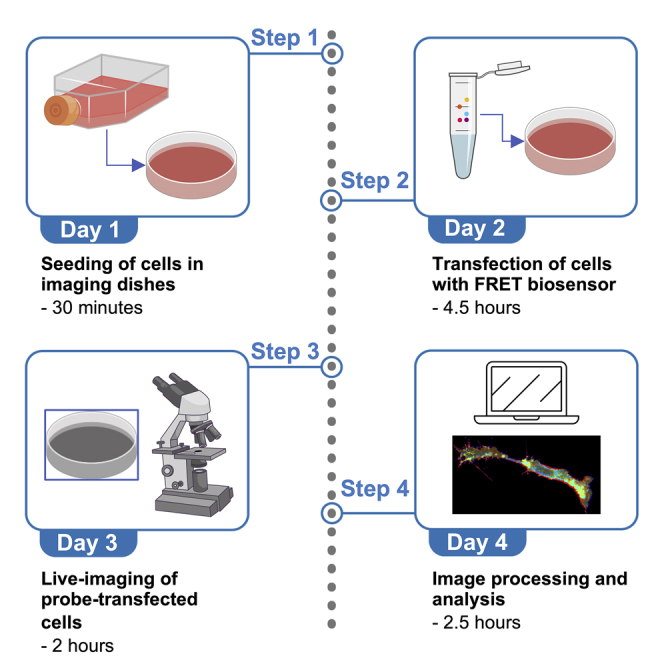

## Before You Begin

**Timing: 6–7 days**

1. Start a culture of low passage HMC3 cells and passage them every time they reach 80%–90% confluency (assessed by visually inspecting cells in an inverted phase-contrast microscope).

**Figure 1:**
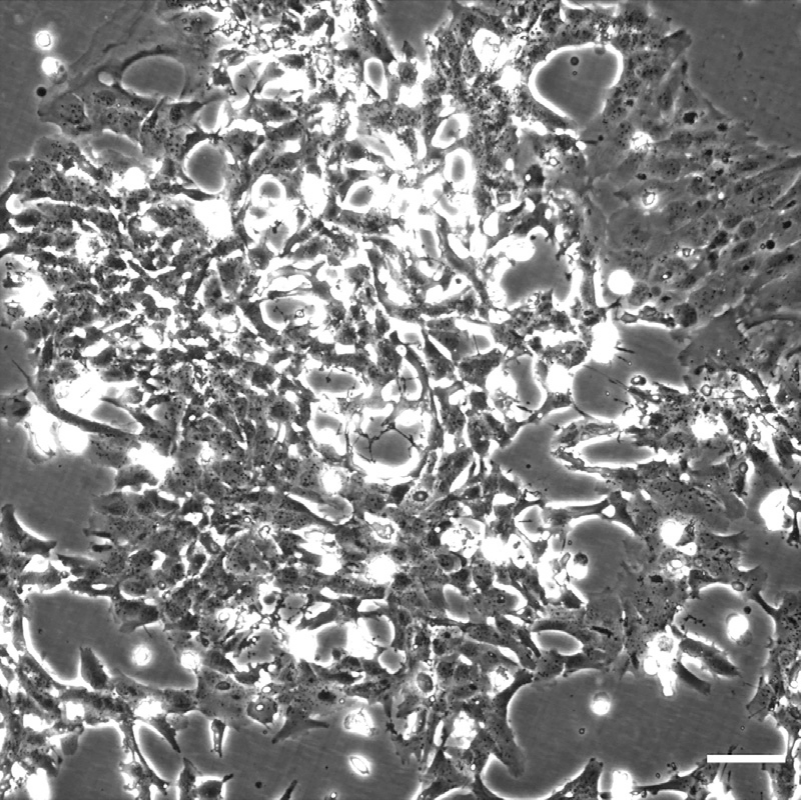
Overconfluent Culture of HMC3 Microglia, Seen in a Phase-Contrast Microscope Notice the clump of aggregated cells and cells growing on top of each other. These are clear indications that overly confluency has been reached and that cells should be discarded. Scale bar: 100 μm.

**CRITICAL:** Do not use cells from cultures that are under-confluent (below 40%) or overly confluent (above 95%), as this will negatively influence transfection efficiency later on. If these cells are allowed to proliferate beyond confluency, they start growing on top of each other and generate big clumps (Figure 1). Splitting cells in these conditions will lead to low growth and survival, severely decreasing transfection efficiency (in the next step).

***Note:*** High passage numbers (from 40 upwards) tend to decrease the efficiency of transfection. We typically start cultures from passages up to 20, which allows for several rounds of expansion and collection before going back to a lower batch number. To facilitate cell recovery after thawing, we initially seed 5×10^5^ cells in a T25 flask (density of 2×10^4^ cells/cm^2^).

***Note:*** Planning for 2–3 passages a week by controlling cells' density or maintaining several plates at different confluencies can ensure material availability.

***Note:*** We use supplemented DMEM (plus 10% FBS, 1% Pen/Strep) as a medium, but RPMI also gives good results.

***Note:*** We incubate the cells at 37°C in an atmosphere of 5% CO_2_.

2. Obtain and quantify the plasmid DNA encoding the FRET probe of interest.

***Note:*** The plasmid used in this protocol was a kind gift from Professor Matsuda (see Key Resources Table). We have used many other probes from Addgene, an open platform for deposition, and sharing ready-to-use plasmids (check https://help.addgene.org for information on ordering).

***Note:*** High-quality plasmid DNA increases the efficiency of transfection, so, if available, use a plasmid DNA purification kit (but not mandatory).

***Note:*** We have successfully used probes from stocks at high (above 1 μg/μL) and low (below 200 ng/μL) concentration. When quantifying the DNA, pay attention to the 260 nm/280 nm and 260 nm/230 nm ratios. For reliable transfection results, we recommend using DNA with values 1.7 - 1.9 for 260 nm/280 nm and 2.0 – 2.2 for 260 nm/230 nm.

## Key Resources Table

**Table:** 

REAGENT or RESOURCE	SOURCE	IDENTIFIER
**Chemicals, Peptides, and Recombinant Proteins**		

Amyloid β Protein Fragment 1–42	Sigma-Aldrich	Cat#A9810; CAS Number 107761-42-2
NaCl	Tocris	Cat# **3146**; CAS Number 7647-14-5
KCl	Tocris	Cat# **3147**; CAS Number 7447-40-7
HEPES	Sigma-Aldrich	Cat#**H3375;** CAS Number 7365-45-9
DMEM, high glucose, GlutaMAX™ Supplement, pyruvate	ThermoFisher	Cat#31966047
Penicillin-Streptomycin (10,000 U/mL)	ThermoFisher	Cat#15140122
Fetal Bovine Serum, qualified, Brazil	ThermoFisher	Cat#10270106
D-(+)-Glucose	Tocris	Cat# **5504**; CAS Number 50-99-7
MgCl_2_·6H_2_O	Sigma-Aldrich	Cat# 442611-M; CAS Number 7791-18-6
CaCl_2_·2H_2_O	Sigma-Aldrich	Cat#C8106; CAS Number 10035-04-8

**Deposited Data**		

Raw image series	This work	https://doi.org/10.17632/y6z2zmsx4f.1

**Experimental Models: Cell Lines**		

HMC3 microglia	ATCC	Cat#CRL-3304; RRID:CVCL_II76

**Recombinant DNA**		

Raichu-RhoA	Michiyuki Matsuda; from Yoshizaki et al., 2003 [PMID:12860967]	N/A

**Software and Algorithms**		

FIJI	PMID:22743772	RRID:SCR_002285
Adobe Illustrator	http://www.adobe.com/products/illustrator.html	RRID:SCR_010279
Precision FRET (PFRET) data processing package for ImageJ	Amassi Perisamy	https://lvg.virginia.edu/digital-downloads/pfret-data-processing-software
R Project for Statistical Computing	http://www.r-project.org/	**RRID:SCR_001905**

**Other**		

jetPRIME®	Polyplus	Cat#114-15
μ-Dish 35 mm, high (microscope imaging dish)	Ibidi	Cat#81156
Microscope objective (HCX PL APO CS 63×/1.30 GLYC 21°C)	Leica Microsystems	NA
Leica DMI6000	Leica Microsystems	NA
Leica EL6000 alignment-free external light source with attenuator and fast shutter	Leica Microsystems	NA
**CFP/YFP/GFP/MRFP External filter sets**	Leica Microsystems	NA
ORCA-Flash4.0 LT+ Digital CMOS camera	Hamamatsu	Cat#C11440-42U30

## Materials and Equipment

**Recipe:** Imaging solution.

• Preparation:
  ○ Weigh NaCl, KCl, HEPES, and glucose and solubilize in MilliQ water;
  ○ Dilute MgCl_2_ (stock 2.4 M) and CaCl_2_ (stock 4 M) and add dropwise:
    • Do not add MgCl_2_ and CaCl_2_ powder to the solution;
  ○ Adjust the pH to 7.4 using NaOH 10 M;
  ○ Complete the solution by adding MilliQ water until the final volume of 100 mL is reached.

**Table:** 

Reagent	Final Concentration	Amount
NaCl	140 mM	820 mg
KCl	5 mM	37 mg
HEPES	20 mM	476 mg
Glucose	4 mM	72 mg
MgCl_2_ (stock solution at 2.4 M)	1 mM	41.6 μL
CaCl_2_ (stock solution at 4 M)	2 mM	50 μL
ddH_2_O	n/a	98.5 mL
**Total**	**n/a**	**100 mL**

## Step-By-Step Method Details

### Seeding of Cells in Imaging Dishes (Day 1)

**Timing: 30 min**

Cultivate cells in an imaging-compatible dish.

1. Detach cells from culture:
  a. Aspirate the medium from the flask;
  b. Add 3 mL PBS (Ca^2+^/Mg^2+^ free) to the flask and gently swirl it to remove traces of serum from the culture medium;
  c. Aspirate the PBS from the flask;
  d. Add 500 μL trypsin 0.5% and tilt the flask up and down and from side to side to ensure coverage of the whole flask surface;
  e. Incubate cells at 37°C for 5 min to allow complete detachment from the flask (cells should be round and free-floating under the microscope);
2. Collect the detached cells:
  a. Add 5 mL supplemented medium to the flask using a serological pipette and use that volume to gently pipette up and down along the flask surface to collect all the detached cells. Transfer the cell suspension to a 15 mL falcon tube;
  b. Centrifuge the cells for 5 min, 400 × *g* at 22°C–25°C;
  c. Discard the supernatant and gently resuspend the cells in 1 mL supplemented medium;
3. Count the cells in suspension using a cell counter or a Neubauer chamber.
4. Seed the cells in the imaging dish:
  a. Add 2 mL supplemented medium to the imaging dish;
  b. Add 2.5×10^4^ cells to this dish (for an area of 35 mm - approximately 7×10^3^ cells/cm^2^) and, using a 1 mL micropipette, gently aspirate up and down the cell suspension in the dish, taking care not to introduce bubbles, to avoid later clustering of attached cells;
  c. Incubate the cells 12–16 h at 37°C (5% CO_2_) to allow attachment and spreading;

***Optional:*** Regarding step 4c. The 12–16 h incubation of cells after seeding is not mandatory before transfection. We tend to see better transfection results when cells are allowed more recovery time post-seeding. If necessary, cells can be transfected as soon as they become attached and spread (at least 2 h post-seeding).

***Note:*** Regarding cell cultivation, any imaging dish with optically clear glass or plastic coverslips can be used without the need for extra cleaning. We recommend #1.5 coverslips to ensure less variance in thickness. We use the Ibidi μ-Dish 35 mm with polymer coverslip bottom for imaging. Dishes with glass coverslip bottoms can also be used with good imaging results. However, we have observed that these cells prefer plastic surfaces (increased growth and survival).

***Note:*** Regarding step 1. The volumes indicated refer to cells seeded in T25 flasks (which we usually use to culture the cells). For bigger containers, scale up accordingly.

### Transfection of Cells with FRET Biosensor (Day 2)

**Timing: 4.5 h**

Transfect cells with a plasmid encoding a FRET probe (CFP/YFP FRET pairs in this example, but can be adapted to other FRET pairs, as appropriate) and incubate for 16–20 h to allow proper biosensor expression.

5. Prepare the plasmid-containing lipid particles for delivery:
  a. For every imaging dish containing cells to be transfected, prepare a 1.5 mL tube with 200 μL JetPrime buffer.
  b. Pipette 900 ng plasmid DNA into this tube and briefly vortex it. (Troubleshooting)
  c. Add 1.8 μL JetPrime transfection reagent to the tube (with the buffer and DNA) and immediately vortex it for 10 s;
  d. Incubate the mixture at 22°C–25°C for 10 min;
6. Transfect the cells and allow particle-cell interaction:
  a. Aspirate the medium from the imaging dish containing the seeded cells;
  b. Add 2 mL of fresh supplemented medium to it;
  c. Use a micropipette to add, dropwise, the entire volume of the plasmid-containing particles (prepared in step 5d) to the dish;
  d. Incubate cells at 37°C (5% CO_2_) for 4 h;
7. Replace the medium:
  a. Aspirate the medium from the imaging dish;
  b. Add 2 mL fresh supplemented medium;
8. Allow probe expression:
  a. Incubate the cells at 37°C (5% CO_2_) for 16–20 h.

**CRITICAL:** Related to step 5b. Never transfect more than 1 μg DNA per plasmid per dish. When using higher amounts, we have observed that probe expression and intracellular distribution are compromised, invalidating the imaging of cells.

**CRITICAL:** Do not skip step 7. Although cells are not overly damaged if the medium is not refreshed, they will be worse for imaging.

**CRITICAL:** Do not shorten the incubation period in step 8a. Proper expression and maturation of the FRET probe require this period. Although different probes can have different maturation periods, a bare-minimum of 12–15 h is necessary for achieving decent imaging.

***Note:*** The ratio of DNA to transfection reagent we use is 1:2, following the manufacturer's instructions (i.e., for every 1 μg plasmid DNA used in step 5b, we add 2 μL of JetPrime transfection reagent).

***Note:*** We use JetPrime transfection reagent because, in our hands, it achieves the best transfection efficiency. However, other lipid-mediated or classical transfection reagents can be used instead (with varying degrees of effectiveness).

### Live Imaging of Probe-Transfected Cells (Day 3)

**Timing: 2 h**

Acquire images of cells transfected with the FRET probe of interest in an inverted fluorescence microscope with appropriate filter sets for CFP-YFP FRET. (Troubleshooting)

***Optional:*** Before beginning any experiment, first inspect if cells are transfected and if probe expression is adequate (inspection should be quick to avoid bleaching the cells) - use any fluorescence microscope with standard FITC or GFP dichroics.

**Figure 2:**
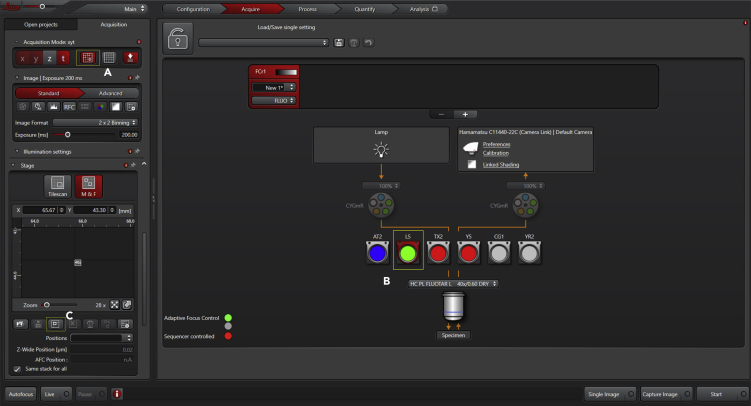
Acquisition Panel with Imaging Settings (Part 1) Activate the mark-and-find option (A) and use the GFP filter (B) to select cells with high expression of FRET probe. Mark the desired positions by adding them one by one (C).
  c. Set up software imaging parameters, exposure time, filter combination, binning adjustment (if applicable), focus and time-lapse routine (Figures 3, 4, and 5);
    i. Light intensity: We use a Leica EL6000 alignment-free external light source at 80% power; (Troubleshooting)
    ii. Exposure: Set exposure in the LAS AF software. Here, pixel intensity values (from 0 to 65535 – 16-bit range) should be at least 800 and a maximum 25000. Such intensity values (using standard CFP/YFP FRET pair under the specified Leica FRET dichroics [CFP ex: BP 427/10; CFP em: BP 472/30; YFP em: BP 542/27], with a high numerical aperture objective, and with the ORCA-Flash4.0 LT+ Digital CMOS camera) are achieved with an exposure time between 50–200 ms — begin with 50 ms and, if required, gradually increase the exposure (up to 200 ms) to reach the minimum pixel intensity value;
    iii. Filter combination:
      - Set DONOR channel – light path to CFP excitation and collect CFP fluorescence; (Troubleshooting)
      - Set ACCEPTOR (FRET) channel – light path to CFP excitation and collect YFP fluorescence;
    iv. Focus mode: Set the focus to adaptative mode (we can record microglia only for short periods – 5/10 min – without using the adaptative focus mode);
    v. Time-lapse routine: Set the time interval between image sequences and the recording interval;

9. Bring cultures to a laminar flow hood:
  a. If using individual dishes, use one at a time;
10. Remove the medium:
  a. Aspirate the medium from the imaging dish;
  b. Add 2 mL saline (PBS or HBSS) - add the solution gently;
  c. Repeat at least two times;
  d. Add 2 mL of imaging solution (see Materials and Equipment for recipe), pre-warmed.
11. Incubate cells with imaging solution:
  a. Allow cells to acclimate for 5–10 min at 22°C–25°C;
  b. Depending on the application (for instance, pre-incubation with agonists, antagonists, activators, and blockers), return the cultures to an incubator. For long-term storage, supplement the media to avoid starvation/acidification of the culture;
  c. Take the culture dish to the microscope (the experimenter can image the cells directly from the dish, but we highly recommend not to skip steps 9–11);
12. Set up the microscope for image acquisition:
  a. Turn on the temperature controller and, if adequate, CO_2_/O_2_ regulators (should be performed before - at least 30 min - incubating cells with imaging solution);
  b. Choose the desired microscope objective and bring cells into focus (Figure 2);
    i. Choose cells with high expression of FRET probe using the GFP filter;
    ii. If more than one field of view (or multiple cells) will be simultaneously recorded, mark and find the desired ones;

**Figure 3:**
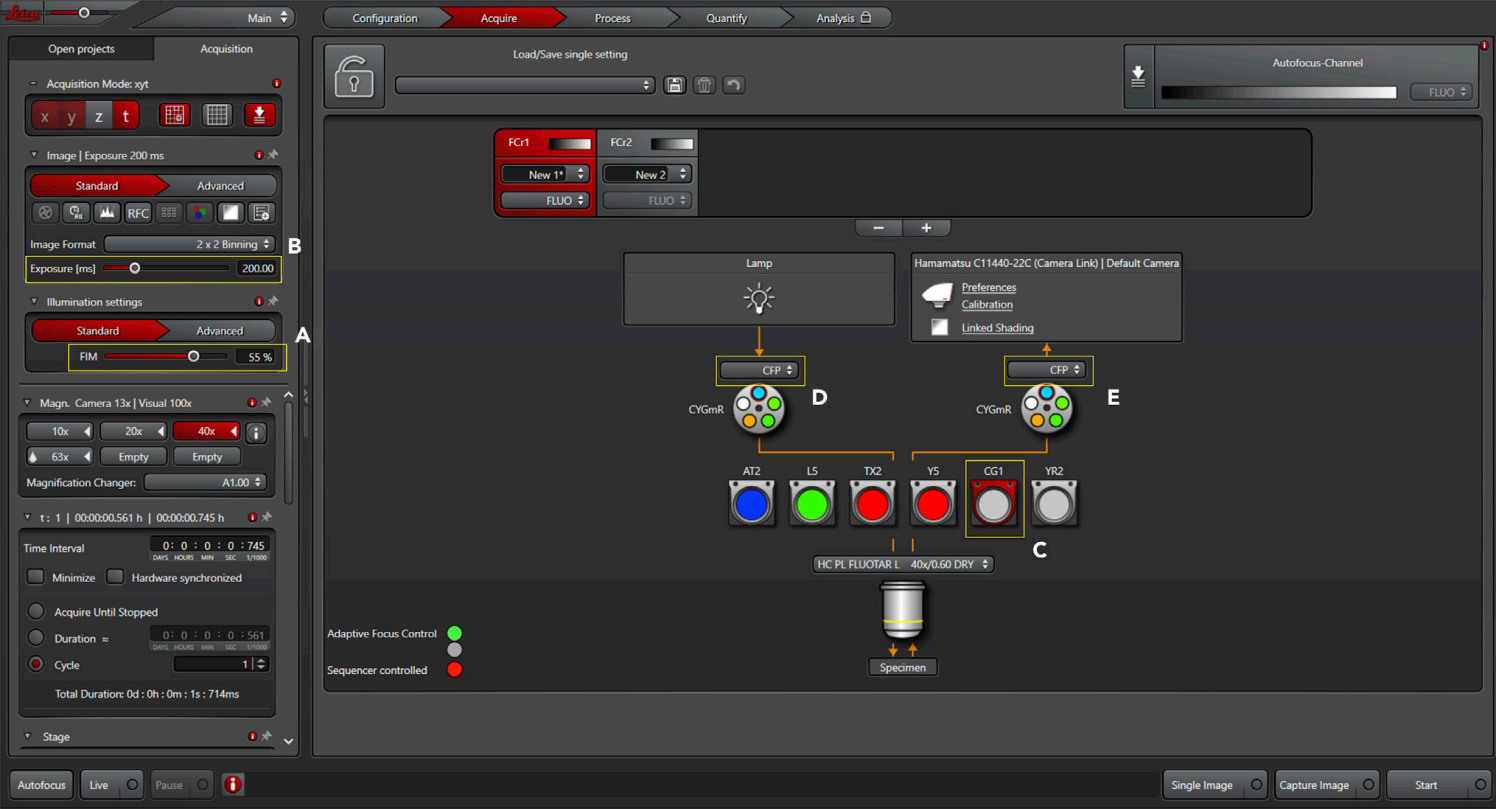
Acquisition Panel with Imaging Settings (Part 2) Once positions have been identified, define illumination intensity (A) and the exposure time (B). Set the acquisition of FRET donor images by selecting the FRET filter (C) and setting the excitation to CFP (D) and emission to CFP (E).

**Figure 4:**
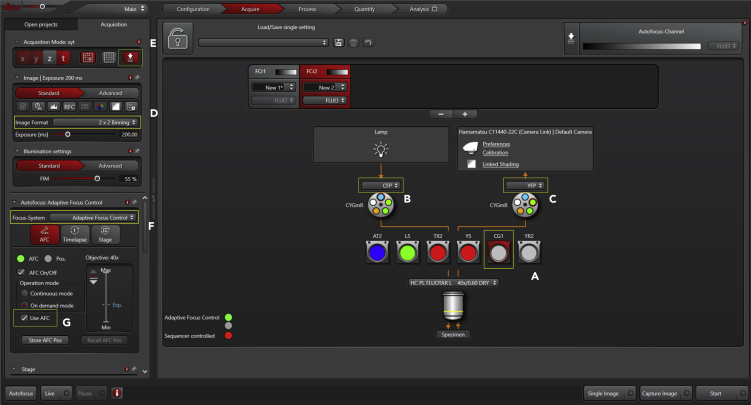
Acquisition Panel with Imaging Settings (Part 3) Set the acquisition of FRET images by selecting the FRET filter (A) and setting the excitation to CFP (B) and emission to YFP (C). If appropriate, define binning (D). Turn the autofocus system on (E) and select Adaptive Focus Control (F). Once cells are in focus, define the AFC point by ticking the option "Use AFC" (G).

**Figure 5:**
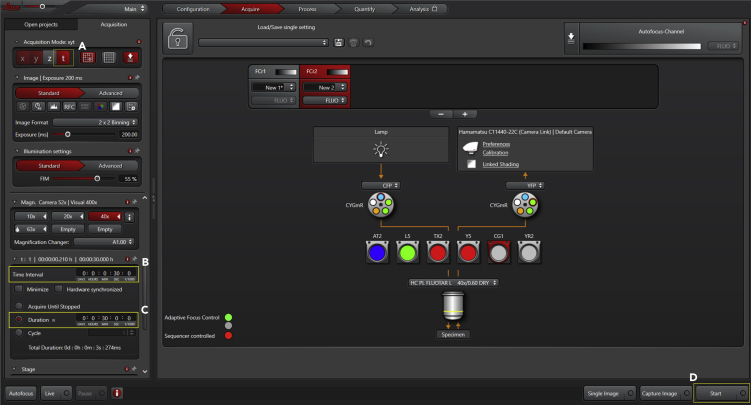
Acquisition Panel with Imaging Settings (Part 4) Activate temporal acquisition of images (A). Set the time-lapse conditions by specifying the frame-rate (B) and the duration of imaging (C). Everything is now ready to start imaging (D).
13. Perform the imaging of selected cells:
  a. In case cells are to be stimulated during imaging acquisition, perform a baseline recording of 5–10 min before adding the stimulus to serve as the internal control for repetitive measures; (Troubleshooting)
  b. After baseline recording, pause the acquisition and apply the solution containing the stimulus; (Troubleshooting)

**CRITICAL:** Do not skip step 10.

**CRITICAL:** Exposure time must be the same for the DONOR and FRET channels; otherwise, the dynamic range of the probe will be disrupted, and analyses will be compromised.

**CRITICAL:** Always use adaptative focus (an in-build "plug and play" routine in the Leica FRET system – see Figures 4E−4G) or similar routines for long-term recordings – Relative to focus mode in step 12c.

**CRITICAL:** Perform experiments at 32°C–35°C (obligatory for long-term recordings) – recordings performed at 22°C–25°C tend to be less reproducible.

**CRITICAL:** Do not use the light source at 100% power – Relative to step 12c.i.

**CRITICAL:** Relative to step 12c.ii. Do not use exposure times higher than 300 ms. Consider using binning if the fluorescence of the FRET probe is too dim. Although applying binning may improve the FRET probe's signal-to-noise ratio, it will also detrimentally affect the image's spatial resolution. With the ORCA-Flash4.0 LT+ Digital CMOS camera, a 2 X 2 binning is a good option to increase the signal-to-noise ratio without losing too much spatial resolution.

***Note:*** Regarding step 13. To improve reproducibility between experiments, the time interval from choosing appropriate cells to beginning the experiment should take less than 30 min.

***Note:*** If adding any solution to the cells during the recording period (for instance, a pharmacological stimulator or inhibitor), start the experiments without using the lid of the culture dish and do not use the mini chamber for gas regulation. For long-term applications, this might be problematic because appropriate pH control requires the use of CO_2_.

***Note:*** If necessary, add phenol red to the imaging solution to visualize media acidification, but this may decrease the signal-to-noise ratio.

### Image Processing and Analysis (Day 4)

**Timing: 2.5 h**

**Figure 6:**
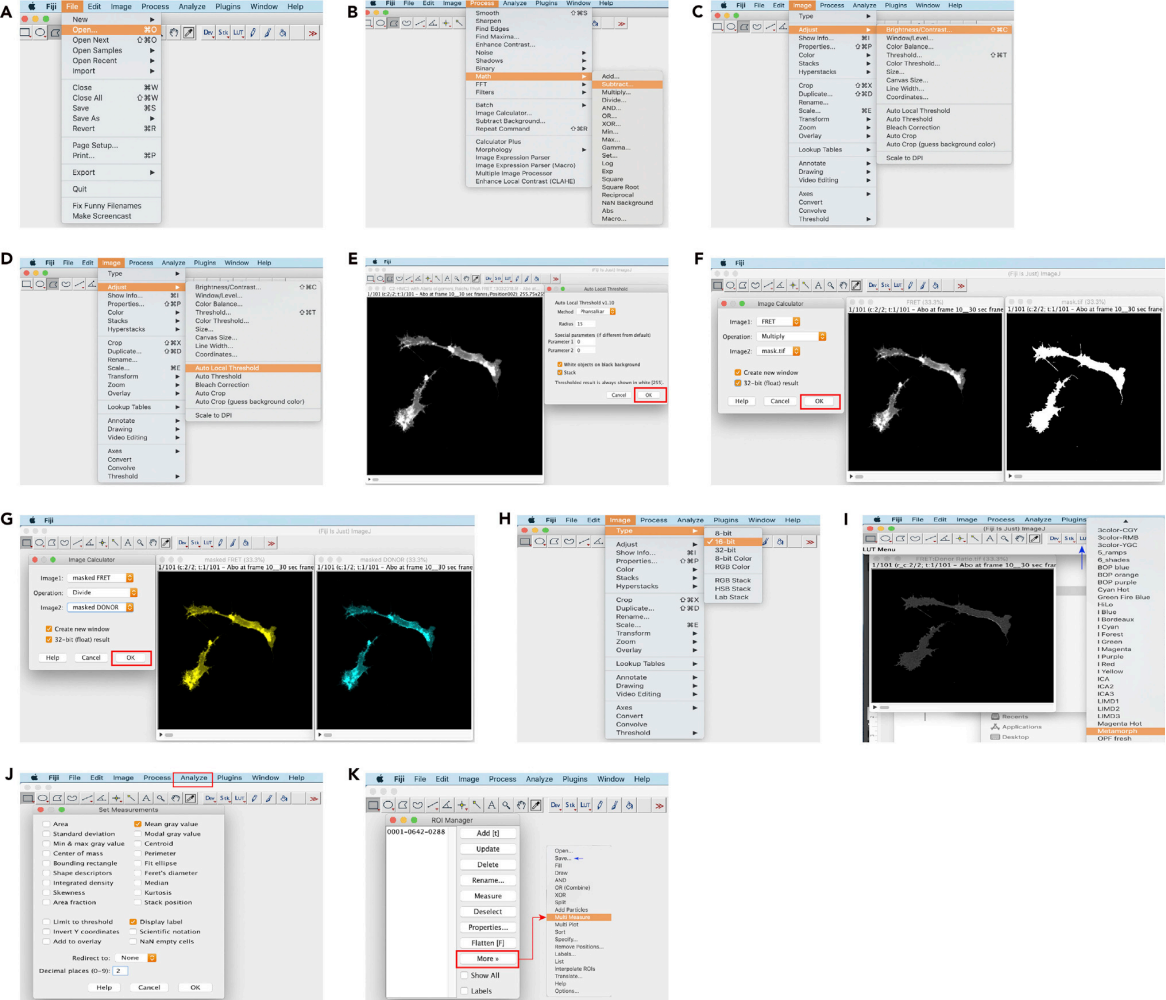
Routine for the Manual Processing of a Live-Cell Imaging CFP/YFP FRET Experiment Manual FRET analysis routine consists of opening the microscope data (A), subtracting the image background (B), adjusting brightness and contrast for the mask (C), thresholding (D) and generating the binary mask (E), masking DONOR and FRET channels (F), producing the ratio image (G), converting the image to 16-bit scale (H), applying a multicolor LUT (I), setting up quantification parameters (J), and quantifying the ROIs using the ROI manager (K).

**Figure 7:**
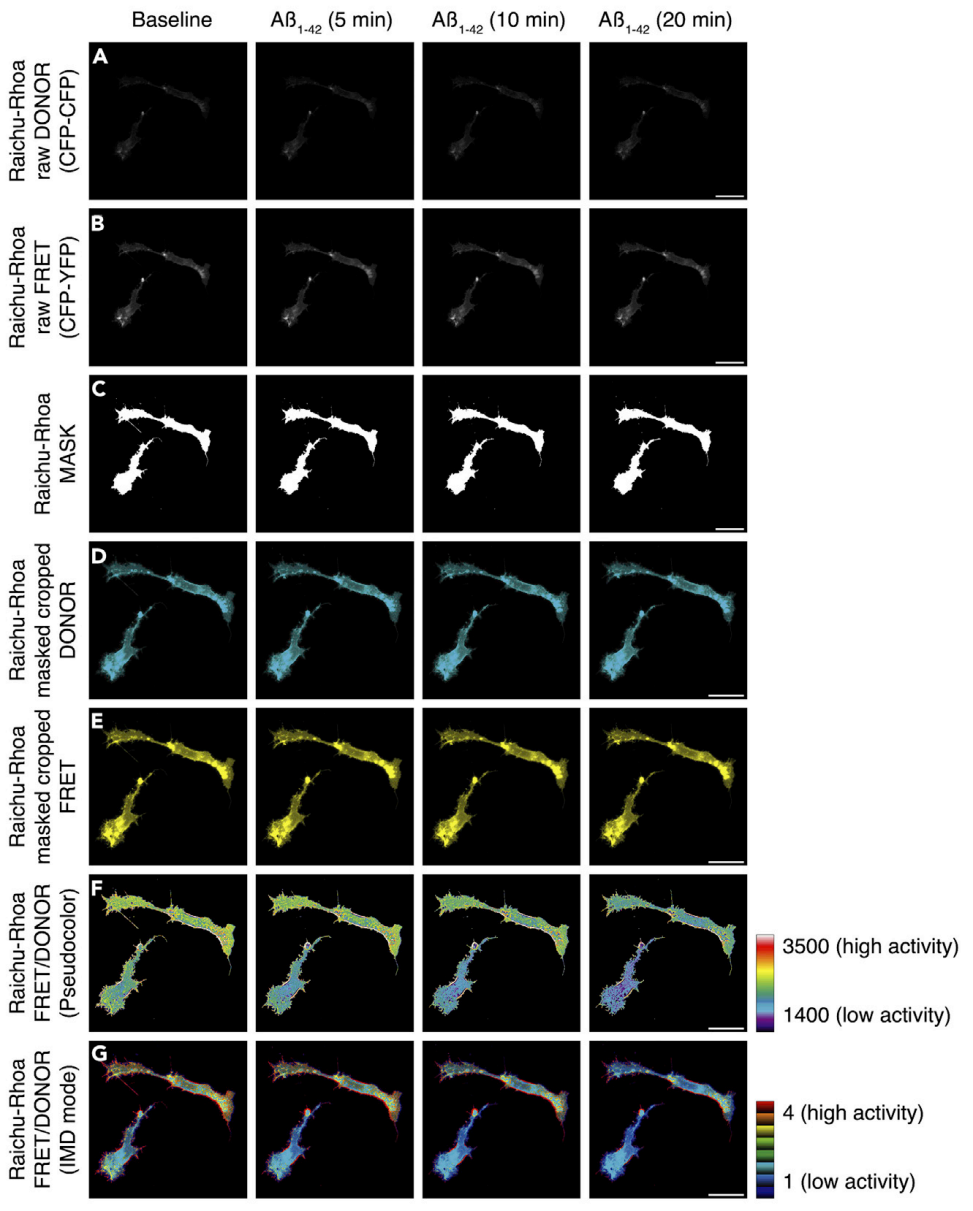
HCM3 Microglia Expressing the RhoA FRET Activity Biosensor Raichu-RhoA Were Recorded in Saline Before (Saline) and upon Exposure to 200 nM Aβ_1-42_ Oligomers Time-lapse images showing DONOR images (A), FRET images (B), binary masks (C), masked DONOR images (D), masked FRET images (E), ratio FRET/DONOR images (F), and IMD images (G). The look-up table ramps indicate the scaling of ratio images in (F) and (G). Scale bars: 20 μm.

Process time-lapses to extract relevant qualitative and quantitative information from image series. The routine (see Figure 6) shows an example of an image series of a CFP/YFP FRET experiment (Figure 7). Troubleshooting

14. Export time-lapse images with metadata; Troubleshooting
15. Open images using FIJI software (Figure 6A);
16. Carefully inspect the time series of DONOR and FRET channels for fluorescence aberrations and photobleaching; Troubleshooting
17. Processing – Here, we outline a completely manual downstream routine; Troubleshooting
  a. Subtract background - DONOR and FRET channels separately:
    i. Draw an ROI in a cell-free zone;
    ii. Measure (shortcut "m") the fluorescence;
    iii. Subtract the values from each corresponding channel (Figure 6B):
      Go to: Process -> Math -> Subtract -> insert corresponding value;
    iv. Adjust brightness and contrast (Figure 6C):
      Go to: Image -> Adjust -> Brightness/Contrast -> Hit “Auto” – **do not** hit “Apply;”
    v. Save DONOR and FRET images as .tiff;
  b. Mask channels:
    i. Duplicate the entire FRET channel stack;
    ii. Threshold the desired ROI (usually a cell of interest in the field) of the duplicated FRET channel stack – (FIJI offers several auto local threshold routines):
      Go to: Image -> Adjust -> Auto Local Threshold (Figure 6D) – 8-bit image required;
    iii. Convert the thresholded ROI into binary (white foreground – black background) image – (this step is automatic if using an auto local threshold routine) – Figure 6E;
    iv. Save the binary stack as .tiff and rename it to "mask;"
  c. Generate masked 32-bit float images of DONOR and FRET channels (Figure 6F):
    i. Go to: Process -> Image calculator -> choose image 1 (DONOR or FRET – repeat for both channels); image 2 (mask); operation = multiply; checkbox in create new window and in 32-bit float;
  d. Rename to "masked DONOR," "masked FRET" and save stacks as .tiff;
  e. Generate a ratio image (Figure 6G):
    i. Go to: Process -> Image calculator -> choose image 1 (masked FRET); image 2 (masked DONOR); operation = divide; checkbox in create new window and in 32-bit float;
  f. Convert ratio image to 16-bit (Figure 6H):
    i. Go to: Image -> Type -> 16-bit, rename to “FRET-DONOR” and save as .tiff;
  g. Apply a gradient LUT (Figure 6I) and adjust image contrast (Figure 6C);
  h. Draw ROIs (user-defined) in cells or structures to be quantified;
    i. Transpose ROI coordinate vectors to the ROI manager – use the shortcut "t;"
  j. Set the measurements to be extracted form ratio images:
    i. Go to: Analyze –> Set measurements (Figure 6J);
  k. Extract quantitative measures using the "multiple measure" function of the ROI manager (Figure 6K) – experimenter may save ROI locations for future reference;
  l. Copy and paste values to a spreadsheet or Save as .csv for further quantitative analyses and statistics;
18. Display representative time series as intensity-modulated display (IMD) images (optional):
  a. Find the IMD FRET plugin at https://doi.org/10.17632/y6z2zmsx4f.1
    i. Download "IMDFret.txt" file;
    ii. In FIJI go to: Plugins -> Macros -> Install -> Open DONOR, FRET and intensity-modulated (IM - duplication of the brightest channel) images -> Run IMDFret plugin;
    iii. Select numerator, denominator, and IM image;
    iv. Adjust the max/min ratio (multiples of 2 are a good option) as well as the max/min intensity;
    v. In the generated color-coded IMD stack, red-yellow represents high FRET, and blue-green represents low FRET - If required, re-adjust brightness and contrast using the sliders;

**CRITICAL:** Do not skip step 16

***Note:*** Regarding step 17a.iii. Alternatively, perform background subtraction using an average value with an in-built FIJI routine:

• Go to: Process -> Subtract background -> try different values of Rolling ball radius;

***Note:*** Regarding step 17k. If subcellular structures to be quantified are moving, it might be needed to register the FRET and DONOR channels before generating the ratio image:

• Go to: Plugins -> Registration -> Register Virtual Stack Slices (https://imagej.net/Register_Virtual_Stack_Slices)

## Expected Outcomes

Here we illustrate an example of the protocol to visualize the small Rho GTPase RhoA activity in HCM3 microglia before and after exposition to Aβ_1–42_ oligomers (Figure 7). The series of images are related to previous work from our group (Socodato et al., 2020). The displayed time series in Figure 7 shows raw images of DONOR configuration (CFP-CFP; Figure 7A), raw images of FRET configuration (CFP-YFP; Figure 7B), a binary mask for the stack (Figure 7C), background-subtracted, masked, and cropped DONOR configuration (CFP-CFP; Figure 7D), background-subtracted, masked and cropped FRET configuration (CFP-YFP; Figure 7E), pseudocolor ratiometric output (FRET/DONOR; Figure 7F), and IMD image (Figure 7G) from HMC3 microglia transfected with the RhoA biosensor Raichu-RhoA (Yoshizaki et al., 2003).

Upon exposure to 200 nM of Aβ_1–42_ oligomers, the anticipated outcome is a global decrease of RhoA activation over time (visualized as a drop in FRET/DONOR ratio in Figure 7F or decreased amounts of red-yellow color in Figure 7G). If extracting quantitative information, inherent fluorescence values can be explored simultaneously in DONOR vs. FRET images or the pseudocolor ratiometric output (Figure 7F).

**CRITICAL:** Do not quantify data using the IMD images; use the ratiometric output images instead (Figure 1C). IMD images are only for illustrative purposes.

***Note:*** For the preparation of Aβ_1–42_ oligomers, refer to (Socodato et al., 2020).

***Note:*** Raw data images for exemplification of routines in this protocol are available at https://doi.org/10.17632/y6z2zmsx4f.1

## Limitations

We describe CFP/YFP FRET on a widefield microscope for whole-cell FRET measurements with a high numerical aperture objective in this protocol. Lack of access to specialized equipment such as the FRET system described herein or a confocal microscope will make the FRET measurements impractical. Finding a FRET biosensor to perform a particular experiment constitutes another limitation. Although there is a growing list of ready-to-use probes (e.g., in public repositories such as Addgene), a biosensor for a specific application may not be available yet. Besides, the development and validation of new biosensors is a complicated and time-consuming endeavor. Low levels of probe expression will result in poor imaging quality, making experiments unreliable. For every case, care must be taken during imaging sessions to ensure that measurements occur with minimum cell perturbation. Inadequate maintenance of environmental conditions between experiments (temperature and pH of imaging medium, for instance) might also compromise the results. If the image quality is not satisfactory after thorough optimization, then the experiment should be discarded, and a different methodology employed.

## Troubleshooting

### Problem 1

Getting near the recommended 1 μg plasmid limit for transfection, but probe expression is still low (step 5b).

### Potential Solution

Depending on the probe used, sometimes transfecting fewer amounts of plasmid achieves better expression results. If necessary, run a plasmid titration experiment from 100 ng to 1 μg to determine the plasmid's best quantity that produces the probe's highest expression.

### Problem 2

One has no access to a tailored, high-end FRET system (Live Imaging of Probe-transfected Cells (Day 3), steps 9–13).

### Potential Solution

A standard confocal microscope equipped with a 405 laser line can be used alternatively to generate satisfactory data in a CFP-YFP FRET experiment.

### Problem 3

The fluorescence light source available in the imaging system is not adjustable (step 12c.i).

### Potential Solution

Try to use light attenuators or neutral density filters. If these are not an option, try to compensate by decreasing the exposure time (might require additional optimization).

### Problem 4

One is experiencing poor signal-to-noise ratio in the DONOR channel (step 12c.iii).

### Potential Solution

Increase light exposure up to 300 ms, then consider applying binning (2 x 2). Note that binning will decrease spatial resolution. If the problem persists, try to optimize the FRET probe's transfection efficiency or even culture the cells in media without phenol red.

### Problem 5

High (≥ 20% of signal intensity) baseline variation (step 13a).

### Potential Solution

Extend the baseline recording period to last 20–30 min. If variation persists, acclimate cells for longer-periods in an incubator before going to the microscope (might require optimizations in the imaging solution).

### Problem 6

Baseline fluorescence is decreasing ≥ 15% over 10 min (step 13b).

### Potential Solution

Cells might be undergoing photobleaching. Consider decreasing light illumination power or light exposure time.

### Problem 7

The cells got out of focus after the application of a stimulus solution (step 13b).

### Potential Solution

Apply stimulus solution very gently and dropwise. Do not immerse the pipette tip into the cell dish containing the imaging solution.

### Problem 8

One has no interest in offline qualitative information or subcellular FRET analyses of time-lapses (Image Processing and Analysis (Day 4), steps 14–18).

### Potential Solution

Use the microscope software to subtract the background and threshold the cell(s). Retrieve the ROI mean fluorescence intensity for the DONOR and FRET channels and export the values to EXCEL. If not saving the time-lapse images and metadata, do not forget to keep a proper and organized track record of experiments.

### Problem 9

Only interested in the overall assessment of endpoint values from time-lapse images (step14).

### Potential Solution

In this case, time-lapse recordings are not necessary; acquire images only of the desired time-points of interest.

### Problem 10

Photobleaching (step 16).

### Potential Solution

• Scenario 1 (optimal): Restrict bleaching in image series as much as possible (optimize light and exposure parameters, record fewer cells per dish or increase the time interval between frames);
• Scenario 2 (less optimal): If bleaching corrections are required, ensure that bleaching removes no more than 25% of overall intensity – Nolan and colleagues developed an excellent package in R for automatic bleaching correction of image series (Nolan et al., 2017);
• Scenario 3 (more severe): Bleaching removed more than 25% of overall image intensity – consider discarding this cell/field;

### Problem 11

Semi-automated processing (step 17).

### Potential Solution

Perform image analyses routine (processing and ratio image generation) using the precision FRET (PFRET) data processing package for ImageJ (https://lvg.virginia.edu/digital-downloads/pfret-data-processing-software)(Elangovan et al., 2003). For an alternative FRET approach, such as the acceptor photobleaching method, the readers may refer to other excellent sources (Wallrabe and Periasamy, 2005, Roy et al., 2008, Roszik et al., 2008, Karpova and McNally, 2006)

## Resource Availability

### Lead Contact

Further information and requests for resources and reagents should be directed to and will be fulfilled by the Lead Contact, Renato Socodato (renato.socodato@ibmc.up.pt).

### Materials Availability

This study did not generate new unique reagents.

### Data and Code Availability

Images to reproduce this protocol have been deposited to Mendeley Data (https://doi.org/10.17632/y6z2zmsx4f.1).
